# Is threat the only modulator of attentional selectivity? Redefining the Easterbrook hypothesis

**DOI:** 10.3389/fpsyg.2014.01020

**Published:** 2014-09-11

**Authors:** Thomas A. Sørensen, Daniel Barratt

**Affiliations:** ^1^Department of Communication and Psychology, Aalborg UniversityAalborg, Denmark; ^2^Department of International Business Communication, Copenhagen Business SchoolCopenhagen, Denmark

**Keywords:** attention, short-term memory, emotion, arousal, Easterbrook hypothesis

A repeated finding in the emotion literature is that threatening stimuli are capable of capturing attention in some way; that is, the observer narrows their attentional focus on the objects of threat in their environment. While this finding makes sense from an evolutionary perspective, a key question is: which particular mechanism is responsible for the modulation of such attentional selectivity? The common interpretation of Easterbrook's (1959) hypothesis suggests that the mechanism in question is *arousal*, although Easterbrook himself described the mechanism as the drive or motivation to withdraw. According to the Yerkes–Dodson's law, the relationship between arousal and performance resembles an inverted U-shape curve with a moderate level of arousal being associated with an optimal level of performance (Yerkes and Dodson, 1908).

Recently, van Steenbergen et al. (2011) have presented a study that seems to challenge Easterbrook's widely accepted hypothesis. Using pictures from the International Affective Picture System as emotional stimuli, pupil dilation as a measure of arousal, and an anti-saccade task as a measure of attentional selectivity, the authors found that both positive and negative pictures produced an increase in arousal whereas only negative pictures produced an increase in attentional selectivity. The authors conclude that arousal is a necessary but not a sufficient condition for attentional selectivity. Although this study presents an interesting challenge to the Easterbrook hypothesis, it does not rule out an alternative interpretation of the underlying mechanisms.

First, we need to determine whether or not arousal is capable of increasing attentional selectivity *independently* of any emotional manipulation. Our laboratory has developed an arousal manipulation which is based on observer expectancy and thus potentially unconfounded by emotional valence (see Vangkilde et al., 2012, Exp. 3; but also see Berlyne, 1966). In a whole and partial report setup, the temporal onset of the stimulus display is varied in accordance with a certain cue (Sørensen et al., 2014). Given that we use a blocked design, we hypothesize that this manipulation modulates the participant's cortical arousal in a tonic rather than a phasic fashion by means of brain arousal systems such as the Reticular Activation System (RAS). Using the Theory of Visual Attention (TVA; Bundesen, 1990) as a theoretical framework, we are able to model the impact of this arousal on specific attentional parameters (Dyrholm et al., 2011). Significantly, we have found that participants in a heightened state of arousal consistently assign higher attentional weights to the target stimuli (*w*) and are better at distinguishing the target stimuli from the distractors (α), in comparison with the low arousal condition. In addition, we have found that as the level of participants' arousal increases the capacity of their visual short-term memory (*K*) tends to decrease (Sørensen and Bundesen, 2011; McAvinue et al., 2012; Sørensen et al., 2014). These results are consistent with both the Easterbrook hypothesis and the Yerkes–Dodson law: for example, raising the level of arousal to a certain point improves the observer's capacity to attend to relevant objects by effectively boosting the salience of the target stimuli. Following the Yerkes–Dodson law, however, too much arousal is a bad thing: if the salience of the target stimuli is already at ceiling, then the only possible effect of adding yet more arousal will be to increase the salience of the distractors, thus leading to an overall decrease in performance.

If arousal is capable of increasing attentional selectivity independently of any emotional manipulation, then why do van Steenbergen and colleagues find a difference between the positive and negative conditions in their study? (Previous studies by, e.g., McNamara and Fisch, 1964, have shown a similar pattern, with negative arousal mediated by threat tending to have a larger impact on performance than positive arousal mediated by monetary reward.) In order to answer this question, we need to turn to the research on visual attention and emotion. Behavioral studies using paradigms such as the flanker task (Fenske and Eastwood, 2003; Barratt and Bundesen, 2012) and the visual search task (Öhman et al., 2001) suggest that threatening stimuli are capable of capturing attention to a greater extent than both positive and neutral stimuli. Neurobiological studies have pinpointed limbic structures such as the amygdala as playing a crucial role in the appraisal of threat (LeDoux, 1998; Öhman, 2005). The amygdala is thought to generate at least three potential outputs of relevance to the current question. First, the amygdala may trigger bodily arousal via the autonomic nervous system, resulting in the increases in heart rate, respiration, and perspiration typically associated with a fear response (and in the pupil dilation measured by van Steenbergen et al.). Second, the amygdala may trigger cortical arousal via various brain arousal systems such as the RAS, thus increasing the general sensitivity of cortical neurons and ensuring that the observer is in a relatively alert state. The key, however, lies in the third and final output. Significantly, evidence suggests that the amygdala may send an attentional weight (*w*) signal to the visual cortex via reciprocal neural connections, thus ensuring that a threatening object in the visual field receives a greater allocation of attentional processing resources (e.g., Sugase et al., 1999).

In principle, then, arousal and attentional selectivity can be separated, although arousal serves to increase the effects of attentional selectivity. We propose that a general increase in cortical arousal modulated via brain arousal systems such as the RAS is sufficient to cause a narrowing of the attentional focus in line with the Easterbrook hypothesis. On top of this, however, the appearance of threatening stimuli in an otherwise neutral environment (cf. McNamara and Fisch, 1964; van Steenbergen et al., 2011) is capable of triggering a specific calculation of attentional weights which results in the faster and more efficient processing of threatening stimuli relative to neutral stimuli. If, for example, the cortical neurons involved in the processing of threatening stimuli are relatively active in comparison with those neurons coding for neutral stimuli, then this activity will be boosted further by a general level of arousal—an engineering “trick” of evolutionary significance (cf. LeDoux, 1998, p. 288).

## Conflict of interest statement

The authors declare that the research was conducted in the absence of any commercial or financial relationships that could be construed as a potential conflict of interest.
